# Ballistocardiogram: Mechanism and Potential for Unobtrusive Cardiovascular Health Monitoring

**DOI:** 10.1038/srep31297

**Published:** 2016-08-09

**Authors:** Chang-Sei Kim, Stephanie L. Ober, M. Sean McMurtry, Barry A. Finegan, Omer T. Inan, Ramakrishna Mukkamala, Jin-Oh Hahn

**Affiliations:** 1 Department of Mechanical Engineering, University of Maryland, College Park, MD, USA; 2 Department of Medicine, University of Alberta, Edmonton, AB, Canada; 3 Department of Anesthesiology and Pain Medicine, University of Alberta, Edmonton, AB, Canada; 4 School of Electrical and Computer Engineering, Georgia Institute of Technology, Atlanta, GA, USA; 5 Department of Electrical and Computer Engineering, Michigan State University, East Lansing, MI, USA

## Abstract

For more than a century, it has been known that the body recoils each time the heart ejects blood into the arteries. These subtle cardiogenic body movements have been measured with increasingly convenient ballistocardiography (BCG) instruments over the years. A typical BCG measurement shows several waves, most notably the “I”, “J”, and “K” waves. However, the mechanism for the genesis of these waves has remained elusive. We formulated a simple mathematical model of the BCG waveform. We showed that the model could predict the BCG waves as well as physiologic timings and amplitudes of the major waves. The validated model reveals that the principal mechanism for the genesis of the BCG waves is blood pressure gradients in the ascending and descending aorta. This new mechanistic insight may be exploited to allow BCG to realize its potential for unobtrusive monitoring and diagnosis of cardiovascular health and disease.

It has long been known that the body moves with each heartbeat^1^. These subtle movements represent the body’s recoil to cardiac expulsion of blood into the arteries. Various “ballistocardiography (BCG)” instruments have been developed (ranging from tables to wearables^2^,^3^,^4^) to measure the periodic, reactionary forces experienced by the body. Figure 1 illustrates an example of a measured BCG waveform for one heartbeat. The example shows several waves such as the “I”, “J”, and “K” waves, which are typical of BCG recordings. Current understanding of the curious BCG waves is based mainly upon empirical correlations with other measurements such as the electrocardiogram, phonocardiogram, and blood pressure (BP) waveform^5^,^6^. However, theoretical efforts to explain the underlying mechanism have been relatively rare. Further, while a few mathematical models of the BCG waveform have been proposed, these models have either failed to reproduce the key BCG waves^7^ or were too complicated to glean any mechanistic insight^8^. Hence, despite increasing interest in BCG^9^, the origin of the BCG waves has remained mysterious. In this study, we formulated and validated a simple mathematical model of the BCG waveform. The model reveals the primary mechanism of the BCG waves as well as their meaning in terms of cardiovascular parameters of clinical significance. This discovery reveals the potential of BCG for unobtrusive monitoring and diagnosis of cardiovascular health and disease.

## Results

### Simple Mathematical Model of the BCG Waveform

**Figure 2:**
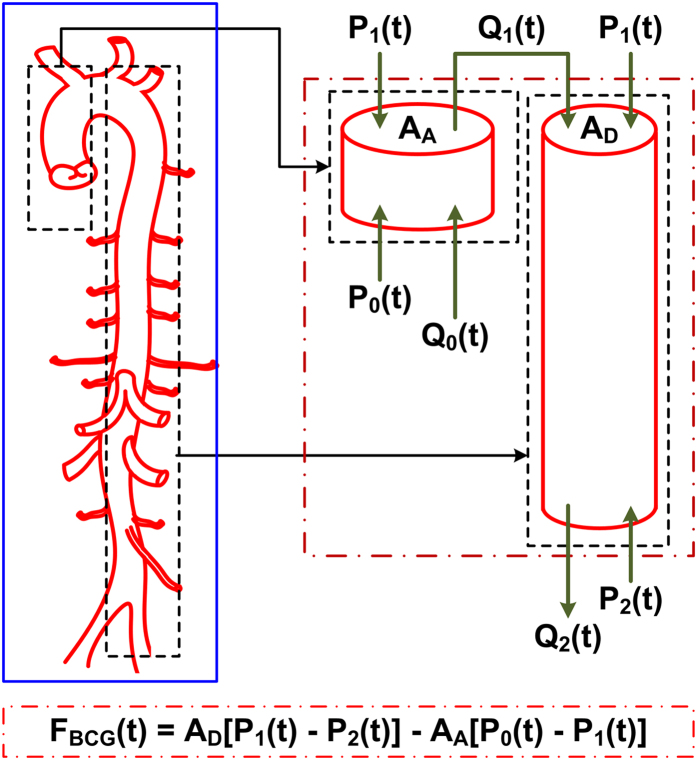
Mathematical model of the BCG waveform. The aorta is simplified as two tubes in cascade. *A*_*A*_ and *A*_*D*_ are the cross-sectional areas of the tubes. The forces acting on blood in each tube are due to the blood pressure (BP; *P(t*)) and volume flow rate (*Q(t*)) waveforms at its inlet and outlet. The subscripts 0, 1 and 2 denote the inlet of the aorta, apex of the aortic arch, and outlet of the aorta. The BCG waveform (*F*_*BCG*_(*t*)) arises as the sum of the forces in both tubes but in the opposite direction. Since the BP terms are much larger than the volume flow rate terms, the model predicts that the principal mechanism for the genesis of the BCG waves is the BP gradients in the ascending and descending aorta.

We mathematically modeled the BCG waveform as an instantaneous force in the head-to-foot direction (*F*_*BCG*_(*t*)) by analyzing the equilibrium of forces exerted on the blood in the main artery of the body, the aorta (see Fig. 2 and Materials and Methods for details). A simple model resulted as follows:


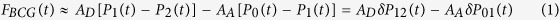


Here, *A*_*A*_ and *A*_*D*_ represent the average cross-sectional areas of the ascending and descending aorta; *P*_0_(*t*) represents BP at the inlet of the ascending aorta; *P*_1_(*t*) represents BP at the outlet of the ascending aorta or inlet of the descending aorta; and *P*_2_(*t*) represents BP at the outlet of the descending aorta. Note that *δP*_01_(*t*) = *P*_0_(*t*)  − *P*_1_(*t*) and *δP*_12_(*t*) = *P*_1_(*t*) − *P*_2_(*t*) constitute the BP gradients in the ascending and descending aorta. Thus, this model predicts that the principal mechanism for the genesis of the BCG waves is BP gradients in the ascending and descending aorta.

### Validity of the BCG Model

**Figure 3:**
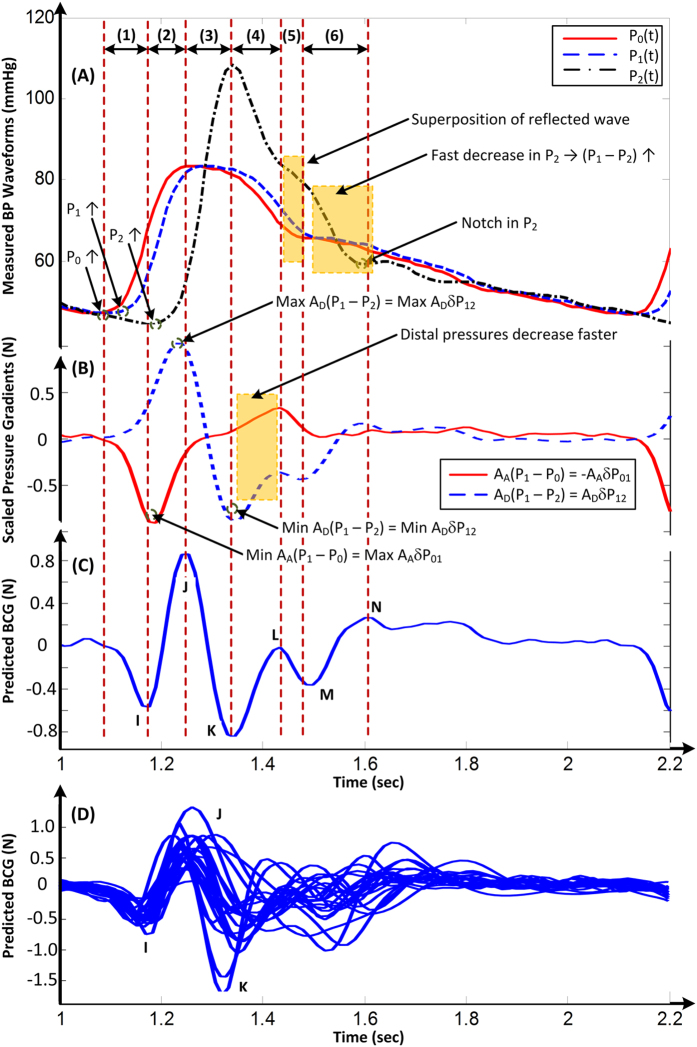
An example of a BCG waveform predicted via the mathematical model of Fig. 2. (**A**) BP waveforms at the inlet of the aorta (*P*_0_), apex of the aortic arch (*P*_1_) and outlet of the aorta (*P*_2_) measured from a human subject. (**B**) Scaled BP gradients in the ascending (*A*_*A*_*δP*_01_) and descending (*A*_*D*_*δP*_12_) aorta calculated from the measured BP waveforms and nominal values for the aortic cross-sectional areas. (**C**) BCG waveform predicted by taking the difference of the scaled BP gradients. (**D**) Predicted BCG waveforms of 21 human subjects.

We tested the validity of the mathematical model by analyzing invasive BP waveforms measured at the inlet and outlet of the aorta from cardiac surgery patients (see Materials and Methods for details). Figure 3A shows an example of the BP waveforms from one patient. We inputted these waveforms, along with nominal values for the aortic cross-sectional areas, into equation (1) to predict the BCG waveform. Figure 3B,C illustrate the resulting BP gradients, scaled by the corresponding cross-sectional areas, and predicted BCG waveform for the patient. The predicted BCG waveforms for the other patients appeared similar (Fig. 3D). The model produced BCG waveforms much like measured BCG waveforms^2^,^5^,^10^,^11^. In particular, it consistently predicted the presence of the major I, J, and K waves (>91% of the patients) and even the minor L, M, and N waves (>83% of the patients). Further, as shown in Table 1, the model predicted physiologic timings and amplitudes for the major waves^12^,^13^. However, the model was unable to predict the minor H wave, which is only sometimes present and could possibly originate from the isovolumic contraction of the heart^8^,^14^.

The predicted BCG waveforms were reasonably robust against modest (±10%) perturbations to the user-selected parameters in the model including the ratio of the aortic cross-sectional areas (*A*_*A*_/*A*_*D*_). In particular, the predicted BCG waveforms exhibited the major I, J, and K waves in >91% of the patients for all different user-selected parameter settings. Further, alterations in the model-predicted timings and amplitudes of these waves were not large, with average absolute alterations of about 0.5% for the timings and 5.5% for the amplitudes with respect to their nominal values.

### Mechanism for the Genesis of the BCG Waves

The mechanism of the BCG waves revealed by the validated model in equation (1) for the patient example in Fig. 3 is as follows. The initial build-up of the I wave is driven by *δP*_01_(*t*), as *P*_0_(*t*) starts to increase in systole while *P*_1_(*t*) is still in diastole (“(1)” in Fig. 3A–C). The I wave peak occurs approximately when *δP*_01_(*t*) is maximal. As *P*_1_(*t*) starts to increase while *P*_2_(*t*) is still in diastole, *δP*_12_(*t*) builds up to cancel and then exceed *δP*_01_(*t*), thereby resulting in the I-J up-stroke (“(2)” in Fig. 3A–C). The J wave peak occurs approximately when *δP*_12_(*t*) is maximal. As *P*_2_(*t*) builds up, *δP*_12_(*t*) decreases, and the J-K down-stroke occurs (“(3)” in Fig. 3A–C). The K wave peak time occurs approximately when *P*_2_(*t*) is maximal or when *δP*_12_(*t*)is minimal. Right after the systolic peak, *P*_2_(*t*) decreases more quickly than *P*_1_(*t*) and results in an increase in *δP*_12_(*t*) (“(4)” in Fig. 3A–C). Meanwhile, *δP*_01_(*t*) exhibits a temporary decrease as *P*_0_(*t*) decreases fast near the dicrotic notch (“(4)” in Fig. 3A–C). These events result in the L wave. Thereafter, *δP*_01_(*t*) increases to zero, while *δP*_12_(*t*) decreases slightly to a local minimum as *P*_2_(*t*) declines more slowly (“(5)” in Fig. 3A–C). These events yield the M wave. The M-N up-stroke is related to the subsequent increase in *δP*_12_(*t*) due to the faster decrease in *P*_2_(*t*) (“(6)” in Fig. 3A–C). Finally, the N wave peak approximately coincides with the time of the local maximum of *δP*_12_(*t*) caused by the diastolic notch appearing in *P*_2_(*t*).

## Discussion

Based on the unveiled mechanism, we can now give the meaning of the timings and amplitudes of the major I, J, and K waves in terms of clinically significant cardiovascular parameters. First, the time of I wave initiation corresponds approximately to the trough or foot of the BP waveform at the inlet of the ascending aorta, while the time of the J wave peak corresponds approximately to the foot of the BP waveform at the outlet of the descending aorta (“(1)” and “(2)” in Fig. 3A–C). Hence, the time interval between the beginning of the I wave and peak of the J wave may represent the aortic pulse transit time, which is a powerful predictor of cardiovascular risk^15^. Second, the amplitude of the J wave corresponds approximately to the aortic pulse pressure (PP = systolic BP − diastolic BP) scaled by the descending aortic cross-sectional area (end of “(2)” in Fig. 3A–C). Since this area may change relatively little^16^, the J wave amplitude may indicate relative changes in the aortic PP, which are often well correlated with relative changes in cardiac stroke volume^17^. Third, the amplitude of the J-K down-stroke corresponds approximately to the peripheral PP scaled by the descending aortic cross-sectional area (“(3)” in Fig. 3A–C). Hence, the ratio of the amplitude of the J-K down-stroke to the amplitude of the J wave may indicate PP amplification, which is another predictor of cardiovascular risk^18^. While we caution that the meaning of the wave features strictly depends on the nature of the BP waveforms (e.g., time delays between waveforms) and aortic cross-sectional areas, the simple meaning provided here could be readily exploited to achieve effective, unobtrusive monitoring and diagnosis of cardiovascular health and disease.

This study has some limitations. First, our patient data did not include BCG measurements. Thus, we were not able to validate the BCG waveform predicted by the model against a BCG waveform measured in the same subject. Future studies should measure both BCG and BP waveforms in the same subjects so that the model may be more rigorously validated. Second, we did not establish the exact location of the outlet of the descending tube in the model. We used the femoral artery as the outlet of the descending tube to validate the model, because it is a reasonable site and femoral BP was available in our patient data. Future studies are needed to ascertain the descending tube outlet location. Note that this location may vary from subject to subject and even within an individual subject due to variations in, for example, arterial stiffness. As a result, such studies may only reveal the descending tube outlet location for an “average” subject. Further note that the model may be relatively insensitive to the exact location of the outlet of the descending tube, because, as the location moves distally, *A*_*D*_ decreases, while the pulse amplitude of *P*_2_(*t*) increases, which may tend to maintain the *A*_*D*_*P*_2_(*t*) term in the model (see equation 1). Thus, an average location may suffice. Third, the insight gained from the model has yet to be translated to actual techniques for estimating clinically significant cardiovascular parameters from the BCG waveform. Such efforts are needed for the findings here to reach healthcare.

## Materials and Methods

### Study Design

The objective of this study was to unveil the mechanism for the genesis of the BCG waveform in order to be able to fully exploit BCG for unobtrusive monitoring and diagnosis of cardiovascular health and disease. First, we developed a simple mathematical model of the BCG waveform. Then, we tested the validity of the model in terms of its ability to predict the BCG waves using data collected from patients. Finally, we elucidated the mechanism of the BCG waves using the validated mathematical model.

### Mathematical Modeling

As shown in Fig. 2, the model approximates the aorta as a short tube (representing the ascending aorta wherein blood moves in the head-ward direction) and a long tube (representing the descending aorta wherein blood moves in the foot-ward direction) connected in cascade. The ascending tube is subject to BP and volume flow rate waveforms at the inlet of the aorta (*P*_0_(*t*) and *Q*_0_(*t*)) and apex of the aortic arch (*P*_1_(*t*) and *Q*_1_(*t*)), while the descending tube is subject to *P*_1_(*t*) and *Q*_1_(*t*) as well as the BP and volume flow rate waveforms at the outlet of the aorta (*P*_2_(*t*) and *Q*_2_(*t*)). Note that, because of pressure wave transmission and reflection in the arteries^19^, all of these waveforms differ in terms of timing, amplitude, and shape (see, e.g., deviations in experimental BP waveforms from different arterial sites in Fig. 3A).

To calculate the forces acting on the blood in the tubes, we considered the blood in each tube as the control volume and made the following simplifying assumptions: (i) blood is homogenous and incompressible; (ii) the cross-sectional area of tube changes little (i.e., the arterial wall is stiff and geometric tapering is small); (iii) blood flow is longitudinal with uniform velocity profile (i.e., inviscid flow). Note that these assumptions are generally well justified based on experimental data^19^. Hence, according to Newton’s second law, the force acting on blood in each tube (*F(t*)) is due to the BP waveforms at its inlet and outlet, which change the blood velocity in the tube, and the volume flow rate waveforms at its inlet and outlet, which change the blood mass in the tube, as follows:


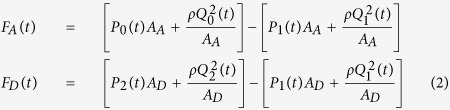


where *ρ* is blood density (which is near that of water), while the subscripts A and D denote the ascending and descending tubes, respectively. Note that a hydrostatic term (*ρgh*, where *g* is gravity and *h* is the vertical distance of the aortic inlet or outlet relative to the heart) could be readily added to the BP terms in this equation when needed (e.g., BCG measurement in standing rather than supine posture). The BCG waveform (*F*_*BCG*_(*t*)) is modeled as the sum of the forces in both tubes but in opposite direction in accordance with Newton’s third law as follows:


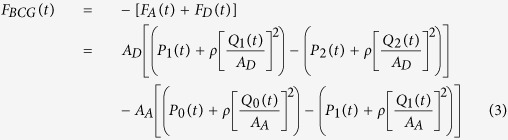


Comparing the relative magnitudes of the BP (*P(t*)) and velocity (*ρ*[*Q(t*)/*A*]^2^) terms in this BCG model suggests that the contribution of the former is much larger than the latter. Indeed, typical aortic blood velocities are around 0.45–0.50 m/s^20^, which yield *ρ*[*Q(t*)/*A*]^2^ values of 1.6–1.9 mmHg, whereas BP is nominally around 100 mmHg. While aortic blood velocity can rise due to either a decrease in aortic cross-sectional area induced by atherosclerosis or an increase in cardiac output induced by exercise or otherwise, its contribution may still be quite small (e.g., <10%)^20^. The BCG model may therefore be simplified to equation (1).

### Model Validation

To test the validity of the mathematical model in equation (1), we used data from human subjects that we previously collected under approval from the University of Alberta Health Research Ethics Board (ID Pro00021889) and informed consent from the patients. Routine standard of care and relevant guidelines were followed. These data, which are described in detail elsewhere^21^, included invasive BP waveforms from the aortic arch and femoral artery of 21 cardiac surgical patients (18 males (age 51–78), 3 females (age 52–79)). We predicted the BCG waveform for each subject as follows. First, we regarded the aortic arch and femoral artery BP waveforms as *P*_1_(*t*) and *P*_2_(*t*) in equation (1), respectively. Second, we advanced the aortic arch BP waveform by a pulse transit time of *τ* = 20 ms to approximate *P*_0_(*t*) in equation (1) as *P*_1_(*t* + *τ*). We arrived at the *τ* value by dividing the typical ascending aortic length (approximately 12 cm) by the corresponding typical pulse wave velocity of old adults (approximately 6.2 m/s)^22^. Third, we estimated the aortic cross-sectional areas *A*_*A*_ and *A*_*D*_ in equation (1) from published anatomical data^23^,^24^. In particular, *A*_*A*_ was estimated as the average of the cross-sectional areas of the ascending aorta (6.78 cm^2^) and aortic arch (5.07 cm^2^), while *A*_*D*_ was estimated as the average of the cross-sectional areas of the thoracic aorta (3.94 cm^2^) and abdominal aorta (1.25 cm^2^). Finally, we predicted the BCG waveform (*F*_*BCG*_(*t*)) by substituting *P*_0_(*t*), *P*_1_(*t*) and *P*_2_(*t*) as well as *A*_*A*_ and *A*_*D*_ into equation (1).

We performed a parametric sensitivity analysis to test the robustness of the predicted BCG waveforms against perturbations to the user-selected parameters in the model of equation (1). The most important user-selected parameters are the pulse transit time (*τ*) and the ratio of the aortic cross-sectional areas (*A*_*A*_/*A*_*D*_). Hence, we varied these parameters by ±10% from their nominal values and examined how the parametric perturbations influenced the shape of the predicted BCG waveforms.

### Statistical analysis

The timings and amplitudes of the BCG waves predicted by the model were presented as mean ± SD.

## Additional Information

**How to cite this article**: Kim, C.-S. *et al*. Ballistocardiogram: Mechanism and Potential for Unobtrusive Cardiovascular Health Monitoring. *Sci. Rep.* **6**, 31297; doi: 10.1038/srep31297 (2016).

## Figures and Tables

**Figure 1:**
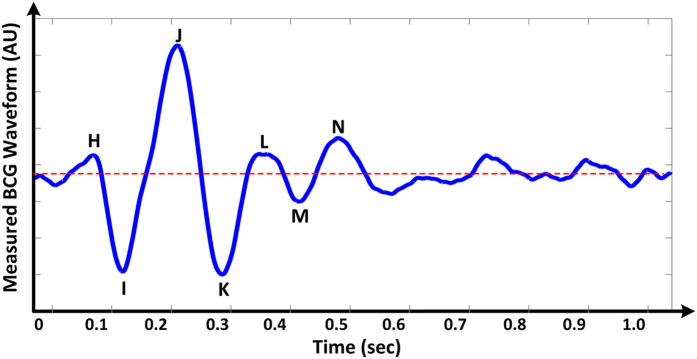
An example of a measured ballistocardiography (BCG) waveform for one heartbeat.

**Table 1:** The timings and amplitudes of the predicted and measured BCG waves; *N* = 21 (mean ± SD).

	Timings			Amplitudes	
	I-J Interval (ms)	J-K Interval (ms)	I-K Interval (ms)	I-IJ Ratio (%)	JK-IJ Ratio (%)
Model	68 ± 11	91 ± 28	158 ± 35	48 ± 11	118 ± 38
13	72	89	161	50.8	129
12	75	88	163	N/A	N/A

Only mean values are shown for the measured waves.
